# Growing media constituents determine the microbial nitrogen conversions in organic growing media for horticulture

**DOI:** 10.1111/1751-7915.12354

**Published:** 2016-03-23

**Authors:** Oliver Grunert, Dirk Reheul, Marie‐Christine Van Labeke, Maaike Perneel, Emma Hernandez‐Sanabria, Siegfried E. Vlaeminck, Nico Boon

**Affiliations:** ^1^ Laboratory of Microbial Ecology and Technology (LabMET)Ghent UniversityCoupure Links 653Gent9000Belgium; ^2^ PeltracomSkaldenstraat 7aDesteldonk9042Belgium; ^3^ Department of Plant ProductionGhent UniversityCoupure Links 653Gent9000Belgium; ^4^ Research Group of Sustainable Energy, Air and Water TechnologyDepartment of Bioscience EngineeringUniversity of AntwerpGroenenborgerlaan 171Antwerpen2020Belgium

## Abstract

Vegetables and fruits are an important part of a healthy food diet, however, the eco‐sustainability of the production of these can still be significantly improved. European farmers and consumers spend an estimated €15.5 billion per year on inorganic fertilizers and the production of N‐fertilizers results in a high carbon footprint. We investigated if fertilizer type and medium constituents determine microbial nitrogen conversions in organic growing media and can be used as a next step towards a more sustainable horticulture. We demonstrated that growing media constituents showed differences in urea hydrolysis, ammonia and nitrite oxidation and in carbon dioxide respiration rate. Interestingly, mixing of the growing media constituents resulted in a stimulation of the function of the microorganisms. The use of organic fertilizer resulted in an increase in *amoA* gene copy number by factor 100 compared to inorganic fertilizers. Our results support our hypothesis that the activity of the functional microbial community with respect to nitrogen turnover in an organic growing medium can be improved by selecting and mixing the appropriate growing media components with each other. These findings contribute to the understanding of the functional microbial community in growing media and its potential role towards a more responsible horticulture.

## Introduction

Vegetables and fruits are fundamental for a healthy diet, with a recommended daily consumption of at least 400 g of both (Who and Consultation, 1990). The eco‐sustainability of their production can be significantly improved. Current systems for growing vegetables employ glasshouses and soilless culture systems (Gruda, 2008). These systems guarantee stable production and high yield of quality products per unit of area, while excluding soil‐borne diseases (Morard, 1995; Grillas *et al*., 2001; Postma, 2009). Growing media are used to support the development of plants and have four main functions: supply roots with nutrients, air, and water, allow for maximum root growth, and physically support the plant. Air and water travel through these pore spaces and water is the medium that carries nutrients that plants need to fuel their growth, and air is needed for root growth and the health of microorganisms.

Growing media are formulated as a blend of different growing media constituents like peat, coir pith, woodfibre, bark, composted materials and usually enriched with lime and inorganic or organic fertilizers to achieve the correct balance of physical and chemical properties. In the Netherlands and Belgium, nearly all vegetables like tomatoes, eggplants, cucumbers and peppers are grown in glasshouses using mineral horticultural growing media (Islam, 2008). Unfortunately mineral growing media lack a diverse and competitive microbial community (Postma, 2009). Environmental and product quality concerns have prompted the search for alternative growing media (Grunert *et al*., 2008; Vaughn *et al*., 2011). Organic growing media and their constituents (Donnan, 1998; Olle *et al*., 2012) exhibits advantages because of their diverse and competitive saprophytic microbial community, which can influence the nutrient status of the root environment (Carlile and Wilson, 1990). In addition, supplementation with liquid effluent from poultry waste, the use of soluble organic fertilizers in a grow bag system and the use of organic fertilizers in combination with peat, perlite and compost made important contributions towards the establishment of sustainable horticulture (Liedl *et al*., 2004; Peet *et al*., 2004; Succop and Newman, 2004; Brentlinger, 2005; Gruda, 2008).

Indeed if we want to use organic‐derived nitrogen in combination with organic growing media, we need to control the conversion of organic forms of nutrients and especially nitrogen into forms that can be taken up by the plants. All heterotrophic soil organisms consume organic materials for energy and carbon and, at the same time, immobilize and mineralize N (Robertson and Groffman, 2007). Ammonium has been viewed as the immediate product of the heterotrophic mineralization, however, also simple soluble organic forms can be taken up by the plants. The next step of the nitrification is the conversion of ammonia (NH_3_) to nitrite (NO_2_^−^), followed by the oxidation of nitrite into nitrate (NO_3_^−^). Ammonia (NH_3_) and nitrite (NO_2_^−^) are mainly converted by chemoautotrophic microorganisms, but heterotrophic bacteria and fungi can produce NO_3_^−^ in acid soils, thereby avoiding ammonification (Hodge *et al*., 2000). Mineralization of the organic‐derived nitrogen in peat is completed by ammonia‐oxidizing archaea (AOA) (Pester *et al*., 2012; Prosser and Nicol, 2012). On the contrary, the low abundance of ammonia‐oxidizing bacteria (AOB) as a consequence of the low NH_3_ concentrations in peat, represents an advantage for the AOA. Previous reports indicated that the activity of the ammonia‐oxidizing *Nitrosomonas* and the nitrite‐oxidizing *Nitrobacter* in peat and peat‐based growing media was positively correlated with the accumulation of ammonia and the addition of fertilizers (Bunt, 2012a,b). Microbial respiration rates increase when the optimal physico–chemical environment is optimal and will potentially lead to high rates of mineralization (Robertson and Groffman, 2007). The C/N ratio of the organic matter determines the availability of the carbon in the material relative to the available nitrogen. Mineralization is favoured over immobilization when the organic fertilizer has a C/N ratio close to the C/N of microorganisms (8:1) (Tisdale *et al*., 1993; Hyvonen *et al*., 1996). Oxygen is required for the oxidation of ammonia and nearly all nitrifiers are obligate aerobes. However the oxygen partial pressure seems to influence the activity, rather than the diversity of AOB (Bodelier *et al*., 1996; Kowalchuk *et al*., 1998; Bollmann and Laanbroek, 2002).

As delivery and production of readily available nutrients derived from microbial activities of growing media constituents and its blends is difficult to predict and to control, it increases our need for investigating the potential of the microbial community associated with growing media to improve the nutrient flow in organic soilless culture systems. In our study, we hypothesized that the activity and the function of the microbial community associated with the growing media constituents is different and that we can obtain a fully well‐balanced nitrifying community (from ammonia till nitrate) when the different functional microbial communities associated with the growing media constituents are blended with each other. We quantified the CO_2_ respiration and nitrification rate of the microbial community associated with different growing media constituents and its blends in batch tests and under controlled, commercial application‐like conditions to develop a fertigation strategy for the growth of tomatoes with organic fertilizers based on organic‐derived nitrogen.

## Results

### CO_2_ respiration rate of different growing media constituents and mixture

Mean rates of soil respiration were all significantly different (*P* = 0.009) between the tested growing media and constituents (Table 1). The Irish peat (10–30 mm) showed the lowest rate of respiration with 16.1 ± 3.1 mg CO_2_‐C kg^−1^ day^−1^, followed by sod peat (10–30 mm) at 25.2 ± 1.5 mg CO_2_‐C kg^−1^ day^−1^ and coconut fibre at 82.6 ± 4.0 mg CO_2_‐C kg^−1^ day^−1^. The blend of the three growing media constituents (40% sod peat 10–30 mm, 40% Irish peat 10–30 mm and 20% coconut fibre, v/v) showed higher carbon respiration rate of 45.9 ± 5.6 mg CO_2_‐C kg^−1^ day^−1^ than calculated based on the volume ratios used in the mixture: 29.4 mg CO_2_‐C kg^−1^ day^−1^. Mixing the growing media constituents resulted in an improved carbon respiration rate (Table 1). The respiration rate of the mineral growing media was equal to the control (−2.3 ± 9.6 mg CO_2_‐C kg^−1^ day^−1^) and the respiration rate of compost was 138.8 ± 20.1 mg CO_2_‐C kg^−1^ day^−1^. The C/N ratio was the highest for the coconut fibre and lowest for the mineral growing medium (Table 1).

**Table 1:** CO_2_ respiration rate of different fresh growing media constituents and a mixture (GB) of the different growing media constituents (*n* = 3)

Growing media and constituents	Carbon dioxide respiration rate (mg CO_2_‐C kg^−1^ day^−1^) (mean ± SD)	C/N ratio
Mineral growing media	−2.33 ± 9.6^a^	3
Compost (green waste)	138.83 ± 20.07^e^	16
Coconut fibre	82.55 ± 3.96^d^	103
Sod peat	25.18 ± 1.46^b^	61
Irish peat	16.05 ± 3.13^a^	49
Mixture GB (20% coconut fibre; 40% Sod peat and 40% Irish peat)	45.86 ± 5.58^c^	ND

Different letters next to the numbers indicate a significant difference (*P* ≤ 0.05). ND, not determined.

**Table 2:** Physico‐chemical analyses of growing medium and constituents (n=1)

	Coconut fibre	Sod peat	Irish peat	Compost	Mixture GB (20% coconut fibre; 40% Sod peat and 40% Irish peat)
Ash content (%)	4	1	1	75	2
Available water (pF1‐pF2) (%)	31	26	9	26	14
Dry matter content (%)	56	53	39	57	50
Shrinkage (%)	23	20	21	23	20
Air content (%)	33	38	39	18	35
Organic matter (%)	96	99	99	25	97
Apparent density (g l^−1^)	98	76	173	481	112
Total pore space (%)	95	96	90	82	93
Humidity (%)	44	47	61	43	50
Water capacity (g/100 DM)	752	731	354	160	424
Water content (pF1) (%)	61	58	51	64	43
Water content (pF2) (%)	30	32	42	38	29
Cellulose (%ads)	40.1	47.4	31.9	8.7	34.9
Hemicellulose (% ads)	13.6	28.5	11.6	5.0	13.1
NDF (% ads)	91.2	93	86.8	22.3	84.9
ADF (%ads)	77.6	64.5	75.1	17.3	71.7
Lignine (%ads)	37.5	17.1	43.2	8.6	36.8
DS (% fresh)	42.7	55	42.2	55.0	47.2
0–1 mm	65	8	16	61	23
1–2 mm	20	3	3	16	6
2–5 mm	13	7	6	16	8
5–8 mm	2	15	10	5	7
8–16 mm	–	45	43	2	35
1716–31 mm	–	21	22	–	17
>31.5 mm	–	–	–	–	–

### Nitrogen transformation rates of the organic growing medium constituents

**Figure 1:**
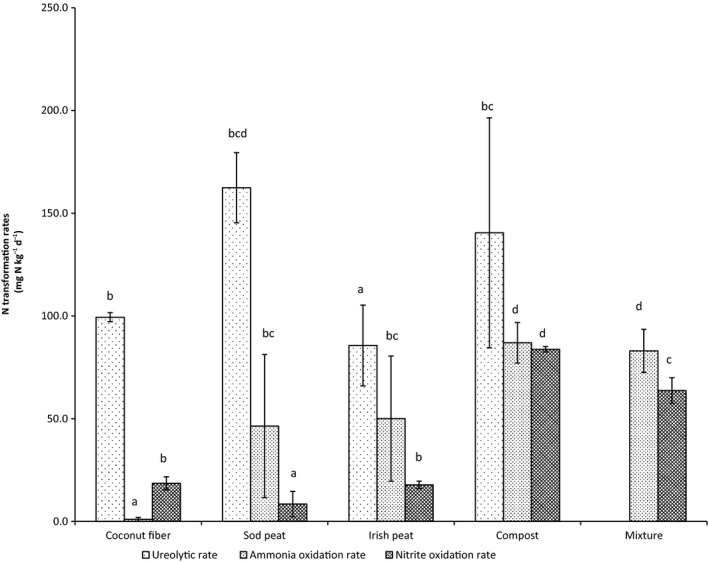
Nitrogen transformation rates by growing media constituent and blends and N treatment. Bars represent standard deviation of triplicate samples. Different letters above the bars indicate a significant difference (*P* ≤ 0.05).

Batch activity tests were performed during 37 days. The ureolysis, the ammonia oxidation and the nitrite oxidation rates are influenced by the growing media constituent and the nitrogen form (urea, ammonium, nitrite and nitrate) used (Fig. 1). The ureolysis (*P* = 0.016), the ammonia oxidation (*P* = 0.015) and the nitrite oxidation rate (*P* = 0.014) were significantly different between growing media constituents. Urea hydrolysis ranged between 162.4 ± 17.1 mg N kg^−1^ day^−1^ and 85.6 ± 19.7 mg N kg^−1^ day^−1^. Sod peat showed the highest and Irish peat the lowest rate of ureolysis. Ammonia oxidation rate ranged between 86.9 ± 9.9 mg N kg^−1^ day^−1^ and 1.0 ± 0.9 mg N kg^−1^ day^−1^ and was the highest in compost and the lowest in coconut fibre. Nitrite oxidation rate was the highest in compost (83.8 ± 1.3 mg N kg^−1^ day^−1^) and lowest in the Sod peat (8.4 ± 6.2 mg N kg^−1^ day^−1^). When growing media constituents were mixed, ammonia oxidation rate increased from 41 to 83 mg N kg^−1^ day^−1^ and nitrite oxidation rate increased from 15 to 63 mg N kg^−1^ day^−1^.

### Development of a fertigation strategy with organic fertilizers suitable for the growth of tomatoes

On the basis of the results obtained in the different batch tests, we developed a fertigation strategy in an in practice simulated environment. First, we tested the mineral (RW) and organic growing media (GB) with an average nitrogen loading rate of 650–700 mg N L^−1^ for the organic fertilizer (OF) and inorganic fertilizer (IF) (Fig. S1). The nitrate concentration in the treatment with IF showed the same tendency for RW and GB and influent and effluent concentration were almost the same for GB and RW. On the contrary, nitrate concentrations in the treatment with OF were 95% lower in comparison to IF, however, increased nitrate concentrations were found in GB after 14–16 days (32 mg NO_3_^−^‐N L^−1^) and 0 mg NO_3_^−^‐N L^−1^) was found in RWOF approximately after 18 days. Moreover, the concentration of the dissolved oxygen (DO) in the IF treatment remained stable (6–8 mg O_2_ L^−1^), whereas DO values in the OF treatment varied between 0 and 2 mg O_2_ L^−1^ (Fig. S2). The treatment with OF resulted in increased amounts of total ammonia nitrogen (TAN; 652 mg N L^−1^ for RWOF and 360 mg N L^−1^ for GBIF). The pH of the RW+OF was about 8.7 and 7.8 for GBOF. Free ammonia levels were 10 times higher for RWOF in comparison to GBOF (Table S4).

In a second experiment, we used the mixture (GB) in combination with OF (Table 7) in order to further optimize the fertigation strategy (Fig. S3). Here, the nitrogen loading rate was gradually decreased from 790 mg N L^−1^ (days 0–29) to 635 mg N L^−1^ (days 30–39), 477 mg N L^−1^ (days 40–48) and 320 mg N L^−1^ (days 49–55). We observed that during the fourth period with the lowest organic nitrogen loading rate (operational day 49–55) on average 22.5% NH_4_^+^ was formed per mg organic nitrogen. This strategy resulted in increased nitrate concentrations with low free ammonia level (0.5 mg N L^−1^) and with the highest DO content (5.05 mg O_2_ L^−1^) in the effluent. Also the pH decreased from 8.2 to 7.6 and the electrical conductivity decreased from 3.7 mS cm^−1^ to 1.3 mS cm^−1^. Blowing extra air into the growing media (GBIF – 0 l min^−1^, GBOF1 – 0 l min^−1^, GBOF2 – 17 l min^−1^ and GBOF3 – 25 l min^−1^) did not increase the ammonification and nitrification rates.

### Comparison of the abundance of the *amoA* gene copy number between growing media, fertilizer and aeration treatments

**Figure 2:**
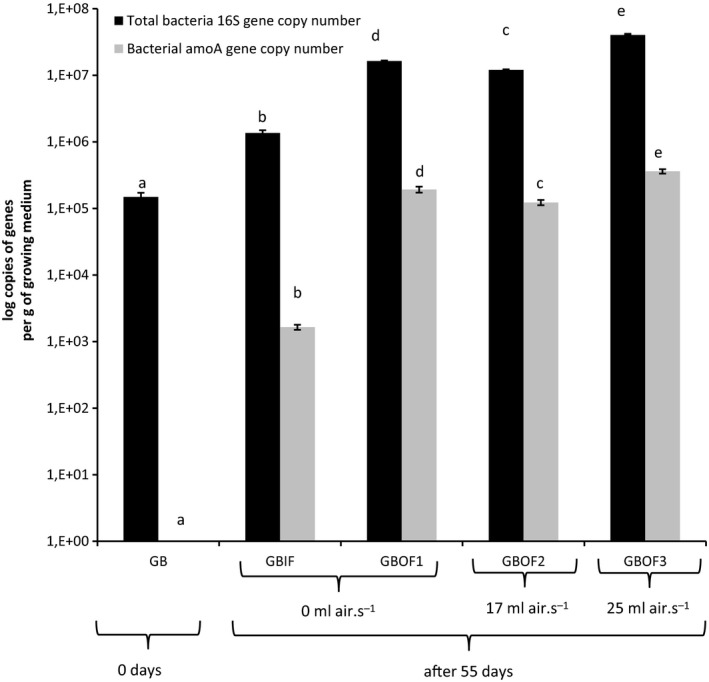
Changes in abundance of bacterial *amoA* genes and total bacteria organic growing media (GB) in combination with inorganic fertilizer (IF) and organic fertilizer (OF). Bars represent standard deviation of triplicate samples. Different letters above the bars indicate a significant difference (*P* < 0.05). GBOF1: organic growing medium with organic fertilizer (0 ml air s^−1^); GBOF2: organic growing medium with organic fertilizer (17 ml air s^−1^); GBOF3: organic growing medium with organic fertilizer (25 ml air s^−1^).

Figure 2 shows the changes in abundance of bacterial *amoA* genes of the organic growing medium treated with organic or inorganic fertilizer. We determined that the AOB *amoA* gene copy number was below detection limit in both fresh growing media at the start of the test. The copy number of the total 16S rRNA genes was below the detection limit (<10^3^ copies g^−1^ of growing medium) for the mineral media (RW). For the mixture we found 1.5 × 10^5^ ± 2.3 × 10^4^ copies per g of the mixture (GB). Growing media with different fertilizer regimes were also analysed after 55 days by qPCR. The percentage of the bacterial copy number of *amoA* gene over de total 16S rRNA gene copy number was 0.12% in the GBIF treatment, 1.17% in the GBOF1, 1.02% in the GBOF2 and 0.90% in the GBOF3 treatment. Blowing air into the growing medium and the use of organic fertilizer had a significant positive effect on the bacterial *amoA* gene copy number (*P* < 0.001) and the total bacteria (*P* < 0.001), however, the ratio *amoA* gene copy number over total 16S rRNA gene copy number remained stable with increasing air flow.

## Discussion

Our study provides evidence that medium constituents, fertilizer type and an adapted fertigation strategy determine microbial nitrogen conversions in organic growing media for horticulture, which greatly strengthen the hypothesis that we can blend functional microbial communities associated with growing media constituents to get a fully nitrifying community in an organic growing medium. This indigenous nitrifying community can be used as a next step towards a more sustainable horticulture, where the delivery and production of organic‐derived nutrients can be predicted and controlled. Significant differences in carbon dioxide respiration rate were found indicating differences in microbial activity between the different constituents. Although the microorganisms associated with the constituents show differences in activity, it is still unclear what their function is. We examined the transformation of different nitrogen species (urea, ammonium, nitrite and nitrate) into ammonium and nitrate, because ammonium and nitrate can be absorbed and assimilated by most plant species.

Carbon respiration showed significant differences between the constituents. Carbon dioxide results from microbial, root and faunal respiration and from non‐biological chemical oxidation (Bunt, 1954). In our case, microbial respiration and activity are important for organic matter mineralization. Moreover, microbial respiration can be used as a measure of community functionality (Bell *et al*., 2005) and increases with the C/N ratio (Spohn, 2015). Higher CO_2_‐C respiration was observed with constituents with higher C/N ratio. Compost however showed the highest carbon respiration rate with the lowest C/N ratio and no respiration was found with RW. Soil respiration is influenced by environmental factors, such as moisture, oxygen supply, carbon content, carbon quality, total pore space, air content, density, pH and microbial populations (Ryan and Law, 2005). In fact, the chemical and physical characteristics were different between the constituents (Table 2, Supplementary Table 2 and Supplementary Table 3) explaining differences in respiration of the different growing media constituents. The molar C:N ratio of the soil microbial biomass at a global scale converges towards 6–8 (Cleveland and Liptzin, 2007; Xu *et al*., 2013). The C:N ratio of soil litter layers is in the range of 12–80 (Berg and McClaugherty, 2003) and the C:N ratio of the used green compost is within this range. The other growing media constituents have lower (RW) or even higher C:N ratios (coconut fibre, sod peat and Irish peat). Indeed microorganisms decomposing organic matter with a higher C:N ratio are confronted with a surplus of C in relation to N and microorganisms confronted with a lower C:N ratio are facing a lack of C in relation to N (Spohn and Chodak, 2015). Compared to other ecosystems, microorganisms presented in growing media and constituents face extreme nutrient perturbations since the C:N ratios of woody plants being part of growing media are extremely high compared to the microbial biomass C:N ratio (Spohn and Chodak, 2015). When growing on N‐poor growing media, microorganisms have not enough N to build up as much biomass as the C concentration would allow resulting in decreased respiration rates in comparison to green compost. It has been argued that N mining, overflow respiration and enzyme inhibition – could explain the observed relationship between qCO_2_ and the litter layer C:N ratio (Spohn and Chodak, 2015). The used compost showed the C:N ratio which was closest to the C:N ratio of soil microbial biomass and had the lowest C:N ratio of the tested organic growing media constituents. This high respiration rate can be explained by the fact that C:N ratios lower than 30:1 allow rapid microbial growth and fast decomposition. As composting proceeds, the C:N ratio gradually decreases from 30:1 to 10–15:1 for the finished product. This occurs because each time that organic compounds are consumed by microorganisms, two‐thirds of the carbon is lost to the atmosphere as CO_2_ gas, while most of the nitrogen is recycled into new microorganisms. Particle size can also affect the availability of carbon and in compost the particle size is very fine in comparison to the other tested constituents. The larger surface area in the compost makes its carbon more readily available for microbial use in comparison to the other constituents. In addition to carbon and nitrogen, adequate phosphorous, sulphur, calcium, and potassium are essential to microbial metabolism, as are trace elements such as magnesium, iron and copper. Normally, these nutrients are not limiting in compost because the compost ingredients provide sufficient quantities for microbial growth. When the constituents were mixed together, the carbon dioxide respiration rate was increased, suggesting changes in the functional properties of the growing medium (Fortin *et al*., 1996). As shown by Robertson and Groffman (2007), respiration and also nitrification rates are optimal at intermediate soil water contents, where the water‐filled pore space, a measure of the soil moisture availability, is about 60%.

Ammonium (NH_4_^+^) and nitrite (NO_2_^−^) are mainly converted by chemoautotrophic microorganisms. Genuinely archaeal and bacterial ammonia oxidizers (AOA and AOB) drive soil nitrification and appear to be ubiquitous, suggesting activity across the pH range investigated. The different growing media constituents have different chemical and physical properties (Table 2, Supplementary Table 2 and Supplementary Table 3). Peat has a pH of about 4.5 and AOB *amoA* genes were not detected in acidic forest soils, tea soils or peat (Stopnišek *et al*., 2010; Yao *et al*., 2011; Isobe *et al*., 2012), because ammonia oxidation in strongly acidic soils is driven by AOA (Zhang *et al*. (2012). Ammonia is a direct substrate for ammonia oxidizers (Suzuki *et al*., 1974), so acidic growing media constituents might be perceived as ammonia‐limited oligotrophic environments, due to exponential ionization of ammonia to ammonium with decreasing pH (De Boer and Kowalchuk, 2001; Zhang *et al*., 2012). The initial ammonium concentrations are relatively low in our two acidic growing media constituents ranging from 2 to 7 mg N l^−1^ growing media, and consequently the ammonia concentrations based on the ionization equilibrium in soil water will be even lower. Coconut fibre on the contrary can be considered as neutrophilic (pH = 6.5) and has a high lignin content. White‐rot fungi are known to break down lignin with the aid of extracellular peroxidase and laccase enzymes. There are also reports of bacteria that can degrade lignin (Bugg *et al*., 2011). Indeed a wide variety of heterotrophic bacteria and fungi can oxidize ammonium. Heterotrophic nitrification appears in some soils and probably also growing media, where the autotrophic nitrifiers are chemically inhibited (Robertson and Groffman, 2007). Ureolytic growth of AOB also occurs at low pH (de Boer and Laanbroek, 1989; Burton and Prosser, 2001), which confirmed the ureolytic activity in sod and Irish peats. The enrichment of the first acidophilic, autotrophic, ammonia oxidizer, *Nitrosotalea devanaterra*, provides an explanation for nitrification in acidic soils (Lehtovirta‐Morley *et al*., 2011). Although NOB prefer a neutral pH‐value as we have in coconut fibre, Hankinson and Schmidt (1988) succeeded in isolating a strain of *Nitrobacter* growing at pH 5.5. Indeed we found that the potential rates for ammonia oxidation and nitrite oxidation were higher for the peat‐based constituents, however, coconut fibre showed higher nitrite oxidation rates (Fig. 1). The results indicate that we have different nitrifying communities in the different growing media constituents. Consequently blending these constituents and also its different nitrifying communities with each other indicates that we potentially have a mix of a heterotrophic and autotrophic nitrifying community in the mixture (GB). We identified in Grunert *et al*. (2016) that Nitrospira and the Nitrosomonadaceae were closely related to GB. In fact, the Actinomycinae, which was highly correlated with the GB in the first time point, had significant correlation with the Nitrospira family. Moreover, Alcaligenaceae has significant correlation with the GB in the third time point. Hence, we identified quite some nitrifying communities that are closely related to GB.

An in practice simulated experiment with the blend showed that the fertigation with IF and OF increased total bacteria and *amoA* copy number per g of growing medium. Also Shen *et al*. (2008) found the highest bacterial *amoA* gene copy numbers in those treatments receiving N fertilizer. Moreover, we saw that *amoA* gene copy number was increased depending on the N fertilizer type used. Use of OF increased the *amoA* copy number 100 times higher (*P* < 0.001) in comparison to IF. The treatment with the highest *amoA* gene copy number had also the highest nitrate formation rates (8% nitrate formed per g organic nitrogen).

Supplement of organic‐derived N needs to be adjusted to the ammonia and nitrite oxidation rates of the microbial community associated with the growing media, resulting in the formation of ammonium and nitrate that can be taken up by the plant (Munch and Velthof, 2006). The increase in nitrate availability in soils and hence growing media is important for plant nutrition. But the microbial community associated with growing medium and the plant may compete for the uptake of N (Hodge *et al*., 2000), especially for NH_4_^+^ (Jackson *et al*., 1989). The optimal growth of tomato roots occurs in soils with a ratio of nitrate to ammonium of 3:1 and is inhibited if the concentration of ammonium is too high (Haynes and Goh, 1978; Glass and Siddiqi, 1995). On the basis of our results of the different batch tests, we developed a fertigation strategy for the growth of tomatoes with OF in combination with an organic growing media. The mineral growing media showed the lowest respiration rates indicating very low or even no microbial activity making it a less optimal growing media in combination with organic fertilizers. In addition to the growing media reactor system (GMRS), that was used to develop a fertigation strategy, there was an accumulation of TAN in the effluent, indicating an incomplete nitrification with a low N‐NO_3_^−^ production efficiency. However, nitrate concentrations were higher in GBOF (8.35%) than in RWOF (0%). Moreover, AOB are inhibited when free ammonia is between 8 and 120 mg N l^−1^ (GBOF: 11.4 mg N l^−1^ and RWOF: 123.9 mg N l^−1^). On the contrary, nitrate production was highest when the organic loading rate was below 315 mg N l^−1^ with acceptable pH (=7), electrical conductivity (1000 μS cm^−1^) and DO (4–5 mg O_2_ l^−1^) levels in combination with the organic growing media. The ratio between ammonium and nitrate was 2:1 and the ammonium concentration was about 70 mg N l^−1^ resulting in ammonium load of 92.4 mg N m^−2^ day^−1^, which is half of the recommended load according to De Kreij and Wever (2005) with 8–10 l water m^−2^ day^−1^.

Due to the scope of the work, the above identified nitrification and respiration rates between different growing media and constituents represent the basis for future research. Additional growing media constituents and organic fertilizers need to be investigated for their potential application in sustainable horticultural settings. Our study provides a comprehensive insight into the physico–chemical interactions, supporting our hypothesis that the bacterial nitrogen turnover is different among horticultural media and its components. In this way, management of the microbial activity can be achieved by selecting and mixing components. However more detailed information is needed on the respiration, and ammonium and nitrite oxidation rate of various blends and constituents under varying growing media moisture contents. These results contribute to the understanding of the functional microbial community and its role towards microbial management of organically derived nitrogen in peat‐based growing medium.

## Material and methods

### Growing medium and constituents

Organic growing medium constituents, that is, sod peat, Irish peat, coconut fibre and compost were separately tested and used as potential inoculum for nitrifying culture as described by Saison *et al*. (2006). Compost was used as a control and is known as a microbial rich growing media constituent (Zeng *et al*., 2013) and the mineral growing medium is known to have no microbial activity (RW, Rock wool, Grotop expert, Grodan). The blend was a mixture of sod peat (40% v/v), Irish peat (40% v/v) and coconut fibre (20% v/v). The blend was fertilized with calcium and magnesium carbonate (Dolokal Extra PG, Ankerpoort, The Netherlands) at 2.5 kg m^−3^ to reach pH(H_2_O) of 5.5. The density of the organic growing medium constituents and blend was determined according to EN 12580. The different constituents and the final growing media were chosen, because of its excellent physico–chemical properties and previous research with plants showed that plants grown in GB a peat‐coconut fibre based growing medium resulted in similar yields like a mineral growing medium (Grunert *et al*., 2008, 2016) in combination with IFs and organic fertilizers. The mineral growing medium was used as a control in the carbon dioxide respiration test and the in practice simulated test (GMRS). Basic chemical and physical properties of the growing media (Table S5) and the constituents were determined and can be found in Table 2, Supplementary Table 2 and Supplementary Table 3.

### Nitrification and respiration rates of organic growing medium constituents

Aerobic batch experiments were performed to measure the intrinsic capacity for urea hydrolysis, ammonia oxidation and nitrite oxidation. For the nitrification batch test, 10 grams of Irish peat, sod peat, coconut fibre and compost resuspended in 100 ml buffer (16.7 g l^−1^ KH_2_PO_4_, 3.3 g l^−1^ K_2_HPO_4_ and 0.6 g l^−1^ NaHCO_3_, pH = 7.1), and incubated in 250‐ml Erlenmeyer flasks, in agitation (120 rpm) at 28°C, under non‐oxygen limiting conditions (DO concentration, ca. 6 mg O_2_ l^−1^, Vlaeminck *et al*. (2007). The RW was not used for the nitrification tests as it showed a carbon respiration comparable with the control, one chamber without growing medium. Batches were supplemented with either urea, NH_4_^+^, NO_2_^−^ or NO_3_^−^ as nitrogen sources and supplemented with NaHCO_3_. The final concentration of the different nutrient types was 100 mg N l^−1^. Flasks were closed with plastic caps to avoid evaporation. Contents in the flasks were allowed to sediment for 10 min before sample collection and pH, EC, DO concentration, and temperature were measured. Two samples of 1 ml of the mixed liquor were collected, filtered through a 0.45‐μm‐pore‐size filter, and analysed for the total N balance (NH_4_^+^‐N, NO_2_^−^‐N and NO_3_^−^‐N). Weight of the flasks was monitored to correct for evaporation.

Respiration of the growing medium constituents was determined following Lundegårdh (1927). In brief, 10 g of fresh soil were mixed with 15 ml of a 0.4 M KOH solution and placed into a sealed headspace chamber (1.5 l) stored in the dark at 28°C for 7 days. As a control, one chamber without growing medium was used. Total mass of trapped CO_2_ was determined by titration of the KOH solutions with 0.02 N HCl. Physical characteristics were measured according to EN13041 (Din, 2012).

### Growing media reactor systems

The lab‐scale GMRS were set up in a dark, temperature‐controlled room (28 ± 2°C). The slabs of organic growing medium (GB) were packed in compostable plastic (EN13432) and had the following dimensions: 0.33 m × 0.2 m × 0.075 m and a volume of 4.42 l ±0.07 l. The slabs of the mineral growing medium (RW) had the same dimensions as GB. Slabs were placed in open plastic containers of 0.39 m × 0.28 m × 0.14 m, and positioned on top of a 3% slope, perforated (Ø 8 mm) at the bottom of the lowest end. Rainwater was used as influent to supplement the different fertilizers and entered at the highest end of the slab, while the effluent of each reactor was collected in a plastic container of 10 l at the lowest end of the slab. The influent flow rate during the experimental period was 1.32 ± 0.01 l day^−1^. Inorganic (IF) and organic nutrient solution (OF) were supplied to the GMRS at fixed time periods every three hours, for 180 s, as described in Fig. S4. Samples of the effluent were collected 0.5 h after the second feeding. The average hydraulic residence time was 0.96 h. The IF system was used as a reference and implemented as in (Kreij, 1997; Sonneveld and Voogt, 2009). The nitrogen loading rate (NH_4_^+^‐N and NO_3_^−^‐N, N‐urea, N_org_) was the same for IF and OF (N_IF_ = N_OF_). The nitrogen load rate was decreased over time in four stages (790 mg N l^−1^ – 630 mg N l^−1^ – 470 mg N l^−1^ – 315 mg N l^−1^). In a first test, the mineral and the organic growing medium were used in combination with IF and OF. In a second test, only the organic growing medium was used in combination with IF and OF. Each treatment had three replicates (*n* = 3). Moreover, air was blown at different velocities into the organic growing medium to provide sufficient oxygen for the nitrification process (GBOF1 = 0 l s^−1^, GBOF2 = 17 ml s^−1^, and GBOF3 = 25 ml s^−1^). The organic fertilizer (OF) was a commercial organic fertilizer (tomato feed from the company Plant Health Cure, Netherlands (7% N – 2% P_2_O_5_ – 3% K_2_O) was used (Table S4). Organic nitrogen contained in this feed consisted mainly of urea (75%, w/w) and other nitrogen sources like amino acids (23%, w/w). The IF had the following composition (Table S4).

### DNA extraction

Total DNA was extracted using physical disruption with the bead beating method from (Hernandez‐Sanabria *et al*., 2012). Cells were lysed in a FastPrep‐96 homogenisator (MP Biomedicals, Illkirch, France) and DNA was precipitated with cold ethanol and resuspended in 30 μl of TAE buffer (10 mM Tris‐HCl, 1 mM EDTA (Ethylenediaminetetraacetic acid) [pH 8.0]). Concentration and quality of DNA were measured based on the absorbance at 260 and 280 nm in a Nanodrop ND 1000 spectrophotometer (NanoDrop Technologies, Wilmington, DE, USA).

### Abundance of ammonia oxidizers

To validate the relationships between total bacteria and AOB in the growing medium, real‐time PCR (qPCR) was performed on a StepOnePlus^™^ real‐time PCR system (Applied Biosystems, Carlsbad, CA, USA). Triplicate samples of a 20‐fold dilution of the DNA samples were analysed to estimate the copy number of the *amoA* of the above bacterial group, following the procedures described by Øvreås *et al*. (1997) and Rotthauwe *et al*. (1997). For the total bacteria (V3 region), the 338F and the 518R primers were used according to Øvreås *et al*. (1997) and for the AOB the amoA‐1F and the amoA‐2R was used according to Rotthauwe *et al*. (1997). The reaction mixture of the 25 μl was prepared by mans of the Thermo Scientific Fermentas PCR master mix (2×) and consisted of 12.5 μl of 2× mastermix, 1 μl of each Primer 5 μl of template DNA and 5.5 μl water. The PCR condition was 95°C for 10 min, followed by 40 cycles of 30 s at 95°C, 60 s at 56°C and 30 s at 72°C. For every cycle, the fluorescence signal capture was at 78°C for 15 s. The qPCR data were represented as copies per ng DNA. The copy numbers of total bacteria were normalized according to the DNA concentrations to express in gene copies per ng DNA. The proportion of the AOB was estimated after dividing the total copy number of 16S rRNA gene by the copy numbers of the targeted gene.

### Physico–chemical analyses of the influent and effluent of the GMRS and of the growing media and constituents

The concentrations of total ammoniacal nitrogen (TAN = NH_4_^+^‐N + NH_3_‐N) and total Kjeldahl‐N (TAN + organic nitrogen) were determined as in (Bremner and Keeney, 1965, A.P.H. Water Environment Federation Association, 1992) Influent and effluent were subjected to determination of nitrite and nitrate concentrations using an ion chromatograph (Metrohm, 930 compact IC flex, Herisau, Switzerland) and DO was measured with a portable meter (Hach Lange Mechelen, Belgium). Chemical oxygen demand was determined using the Photometer Nanocolor 500 D kit, following the manufacturer's instructions (Marcherey‐Nagel, Düren, Germany). Chemical characteristics of the growing media were measured according to (Gabriels *et al*., 1998).

Carbohydrate analyses of the samples was performed as described by Van Soest *et al*. (1991). Neutral detergent fibre (NDF) measures the hemicellulose, cellulose and lignin contained in the plant cells, while the residue remaining after boiling NDF in acid detergent solution is called acid detergent fibre (ADF) and contains the cellulose, lignin and ash present in the samples. Results were expressed as percentage of the absolute dry matter (ADM). C/N ratio, organic matter (OM) content (EN13039 (CEN), total nitrogen (British Standard, 2001) and dry matter content (British Standard, 2000) of the different constituents were determined.

### Statistical analysis

Nitrification and respiration rates and log transformed *amoA* gene copy numbers were compared by one‐way ANOVA followed by All Pairwise Multiple Comparison Tukey to check for quantitative variance between different treatments with a confidence interval of 95%. All analyses were conducted using SPSS version 22.0 (IBM, Armonk, NY, USA), and *P* ≤ 0.05 was considered to be statistically significant. When the data were not normally distributed (*P* < 0.05) and or there was no equal variance (*P* < 0.05), then we used the Kruskal–Wallis one‐way ANOVA on ranks followed by an all pairwise multiple comparison Dunn's test.

## Conflict of interest

The authors whose names are listed immediately below declare none competing commercial interest, affiliation with or involvement in any organization or entity with any financial interest (such as honoraria, educational grants, participation in speakers’ bureaus, membership, employment, consultancies, stock ownership or other equity interest; and expert testimony or patent‐licensing arrangements), or any non‐financial interest (such as personal or professional relationships, affiliations, knowledge or beliefs) in the subject matter or materials discussed in this manuscript.

Oliver Grunert Peltracom/UGent, Belgium; Siegfried E. Vlaeminck Uantwerpen/UGent; Emma Hernandez‐Sanabria UGent, Belgium; Maaike Perneel Peltracom, Belgium; Marie‐Christine Van Labeke UGent, Belgium; Dirk Reheul UGent, Belgium; Nico Boon UGent, Belgium.

## Supporting information


**Fig. S1.** Evolution of the nitrate content in the effluent in an organic and mineral growing with an organic and inorganic fertigation system.

**Fig. S2.** Evolution of the DO levels in the effluent in an organic and mineral growing media with an organic and inorganic fertigation system.

**Fig. S3.** N mineralization in an organic growing medium with an organic‐derived nitrogen source in function of time.
