# Supplementary material for: Growing media constituents determine the microbial nitrogen conversions in organic growing media for horticulture
Source: Microb Biotechnol. 2016 Mar 23;9(3):389–99. doi: 10.1111/1751-7915.12354 (PMC4835575; doi:10.1111/1751-7915.12354)
Supplement: Supplementary file 1 — Fig. S1. Evolution of the nitrate content in the effluent in an organic and mineral growing with an organic and inorganic fertigation system. [file MBT2-9-389-s001.docx]

Days (n) different constituents and the final growing media were chosen, because of its excellent physicochemical properties and previous research with plants showed that plants grown in GB a peat-coco fiber based growing medium resulted in similar yields like a mineral growing medium (Grunert et al., 2008 and Grunert et al, 2015) in combination with inorganic fertilizers.

RWOF

GBOF

GBIF

RWIF

Nitrogen Loading rate (650mg N.L^-1^)

Supplementary Figure 1 : Evolution of the nitrate content in the effluent in an organic and mineral growing with an organic and inorganic fertigation system.

GBIF: organic growing medium with organic fertilizer (100% organic derived nitrogen), RWIF : mineral growing medium with inorganic fertilizer

GBOF: organic growing medium with organic fertilizer (100% organic derived nitrogen) and RWOF: mineral growing medium with organic fertilizer
