# Supplementary material for: Growing media constituents determine the microbial nitrogen conversions in organic growing media for horticulture
Source: Microb Biotechnol. 2016 Mar 23;9(3):389–99. doi: 10.1111/1751-7915.12354 (PMC4835575; doi:10.1111/1751-7915.12354)
Supplement: Supplementary file 2 — Fig. S2. Evolution of the DO levels in the effluent in an organic and mineral growing media with an organic and inorganic fertigation system. [file MBT2-9-389-s002.docx]

GBOF

RWOF

GBIF

RWIF

Supplementary Figure 2 : Evolution of the DO levels in the effluent in an organic and mineral growing media with an organic and inorganic fertigation system. GBIF: organic growing medium with organic fertilizer (100% organic derived nitrogen), RWIF : mineral growing medium with inorganic fertilizer

GBOF: organic growing medium with organic fertilizer (100% organic derived nitrogen) and RWOF: mineral growing medium with organic fertilizer
