# Supplementary material for: Growing media constituents determine the microbial nitrogen conversions in organic growing media for horticulture
Source: Microb Biotechnol. 2016 Mar 23;9(3):389–99. doi: 10.1111/1751-7915.12354 (PMC4835575; doi:10.1111/1751-7915.12354)
Supplement: Supplementary file 3 — Fig. S3. N mineralization in an organic growing medium with an organic‐derived nitrogen source in function of time. [file MBT2-9-389-s003.docx]

Supplementary Figure 3: N mineralization in an organic growing medium with an organic derived nitrogen source in function of time. The used organic nitrogen source is (Total N in) is 100% organic derived nitrogen.
