# Supplementary material for: Growing media constituents determine the microbial nitrogen conversions in organic growing media for horticulture
Source: Microb Biotechnol. 2016 Mar 23;9(3):389–99. doi: 10.1111/1751-7915.12354 (PMC4835575; doi:10.1111/1751-7915.12354)
Supplement: Supplementary file 4 [file MBT2-9-389-s004.docx]

Supplementary Table 1. Chemical composition of the organic (OF) and inorganic nutrient solution (OF) used in the GMRS tests

| **Parameters** | Organic nutrient solution  (OF) | Inorganic nutrient solution  (IF) |
| --- | --- | --- |
| pH | 3,3 | 6,8 |
| Carbon content (mg C. L^-1^) | 17.5 | 0 |
| C/N ratio | 2.18 | 0 |
| Conductivity (µS/cm) | 955 | 2012 |
| Organic-nitrogen (mg N.L^-1^) | 186,0 | 0 |
| Urea-nitrogen (mg N. L^-1^) | 603,0 | 0 |
| Total Ammonia- nitrogen (mg N. L^-1^) | 12,4 | 16,6 |
| Nitrate-nitrogen (mg N. L^-1^) | 1,6 | 288,7 |
| Total-nitrogen (mg N. L^-1^) | 803,0 | 305,4 |
| Phosphorous (mg P. L^-1^) | 90,8 | 55,3 |
| Potassium (mg K. L^-1^) | 112,9 | 371,9 |
| Calcium (mg Ca. L^-1^) | 30,0 | 316,2 |
| Magnesium (mg Mg. L^-1^) | 14,8 | 119,1 |
| Sulphur (mg S. L^-1^l) | 17,2 | 221,3 |
