# Supplementary material for: Growing media constituents determine the microbial nitrogen conversions in organic growing media for horticulture
Source: Microb Biotechnol. 2016 Mar 23;9(3):389–99. doi: 10.1111/1751-7915.12354 (PMC4835575; doi:10.1111/1751-7915.12354)
Supplement: Supplementary file 5 [file MBT2-9-389-s005.docx]

Supplementary Table 2: Chemical analyses of unused mineral and organic growing medium (n=10).

NS= not significantly different. P= (α <0.05)

| Variable | Growing medium | | P value |
| --- | --- | --- | --- |
|  | Mineral growing medium | Organic growing medium |  |
| pH(H2O) | 6.30 ± 0.18 | 4.40 ± 0.10 | < 0.001 |
| Conductivity (µS/cm) | 20.09 ± 1.66 | 90.63 ± 20.99 | < 0.001 |
| Organic nitrogen (mg N/l) | 2.13 ± 2.71 | 0.32 ± 0.55 | NS |
| Total ammonia nitrogen (mg N/l) | 0.51 ± 0.13 | 6.05 ± 0.51 | < 0.001 |
| Nitrite (mg N/l) | 0.00 ± 0.00 | 0.00 ± 0.00 | < 0.001 |
| Nitrate (mg N/l) | 0.00 ± 0.00 | 6.78 ± 5.14 | 0.001 |
| Phosphorous (mg P/l) | 1.43 ± 0.25 | 8.51 ± 1.31 | < 0.001 |
| Potassium (mg K/l) | 6.25 ± 2.12 | 103.75 ± 31.43 | < 0.001 |
| Calcium (mg Ca/l) | 22.25 ± 14.74 | 174.5 ± 28.72 | < 0.001 |
| Magnesium (mg Mg/l) | 6.25 ± 1.77 | 54.75 ± 5.46 | < 0.001 |
| Sulphate (mg S/l) | 7.28 ± 2.01 | 13.85 ± 3.01 | < 0.001 |
| Sodium (mg Na/l) | 9.00 ± 1.29 | 42.75 ± 9.16 | < 0.001 |
| Chloride (mg Cl/l) | 3.42 ± 0.88 | 76.80 ± 24.43 | < 0.001 |
| Iron (mg Fe/l) | 4.13 ± 0.41 | 0.10 ± 0.03 | < 0.001 |
| Manganese (mg Mn/l) | 0.13 ± 0.03 | 0.60 ± 0.11 | < 0.001 |
