# Supplementary material for: Growing media constituents determine the microbial nitrogen conversions in organic growing media for horticulture
Source: Microb Biotechnol. 2016 Mar 23;9(3):389–99. doi: 10.1111/1751-7915.12354 (PMC4835575; doi:10.1111/1751-7915.12354)
Supplement: Supplementary file 6 [file MBT2-9-389-s006.docx]

Supplementary Table 3: Chemical analyses of growing medium constituents.n=1

| Variable | Growing medium constituents | | | | |
| --- | --- | --- | --- | --- | --- |
|  | Coconut fiber | Sod peat | Irish peat | Compost | Mineral wool |
| C/N | 103 | 65 | 49 | 16 | 3 |
| pH(H_2_O) | 5.8 | 4.1 | 4.2 | 8.53 | 6.3 |
| Conductivity (µS/cm) | 579 | 22 | 45 | 559 | 20 |
| Nitrate nitrogen (mg N/l) | 66 | 0 | 0 | 18 | 0 |
| Total ammonia nitrogen (mg N/l) | 10 | 2 | 7 | 47 | 0.5 |
| Phosphorous (mg P/l) | 13 | 11 | 7 | 236 | 1 |
| Potassium (mg K/l) | 688 | 15 | 15 | 1430 | 6 |
| Calcium (mg Ca/l) | 353 | 140 | 158 | 1993 | 22 |
| Magnesium (mg Mg/l) | 65 | 35 | 110 | 248 | 6 |
| Sulphate (mg S/l) | 51 | 63 | 78 | 73 | 7 |
| Sodium (mg Na/l) | 193 | 10 | 28 | 88 | 9 |
| Chloride (mg Cl/l) | 583 | 1 | 10 | 363 | 3 |
| Iron (mg Fe/l) | 0.7 | 0.6 | 0.7 | 4.6 | 4 |
| Manganese (mg Mn/l) | 1.0 | 0.5 | 1.3 | 29.8 | 0.1 |
