# Supplementary material for: Growing media constituents determine the microbial nitrogen conversions in organic growing media for horticulture
Source: Microb Biotechnol. 2016 Mar 23;9(3):389–99. doi: 10.1111/1751-7915.12354 (PMC4835575; doi:10.1111/1751-7915.12354)
Supplement: Supplementary file 7 [file MBT2-9-389-s007.docx]

Supplementary Table 4: Chemical composition of the effluent of the inorganic and organic nutrient solution.

| Treatment | Org N  (mg N.L^-1^) | TAN  (mg N.L^-1^) | N-NO_2_^-^  (mg.L^-1^) | N-NO_3_^-^  (mg N.L^-1^) | Total N  (mg N.L^-1^) | pH | FA  (mg N.L^-1^) | FNA  (mg N.L^-1^) |
| --- | --- | --- | --- | --- | --- | --- | --- | --- |
| RWIF | 0,0 | 66,9 | 0,0 | 631,0 | 697,9 | 7.1 | 0.35 | 0.0000 |
| GBIF | 0,0 | 64,3 | 0,9 | 687,5 | 752,7 | 6.2 | 0.05 | 0.0012 |
| RWOF | 22,0 | 652,9 | 0,0 | 0,0 | 674,9 | 8.7 | 123.9 | 0.0000 |
| GBOF | 5,3 | 360,5 | 21,9 | 51,2 | 438,9 | 7.8 | 11.4 | 0.0008 |
