# Supplementary material for: Growing media constituents determine the microbial nitrogen conversions in organic growing media for horticulture
Source: Microb Biotechnol. 2016 Mar 23;9(3):389–99. doi: 10.1111/1751-7915.12354 (PMC4835575; doi:10.1111/1751-7915.12354)
Supplement: Supplementary file 9 [file MBT2-9-389-s009.docx]

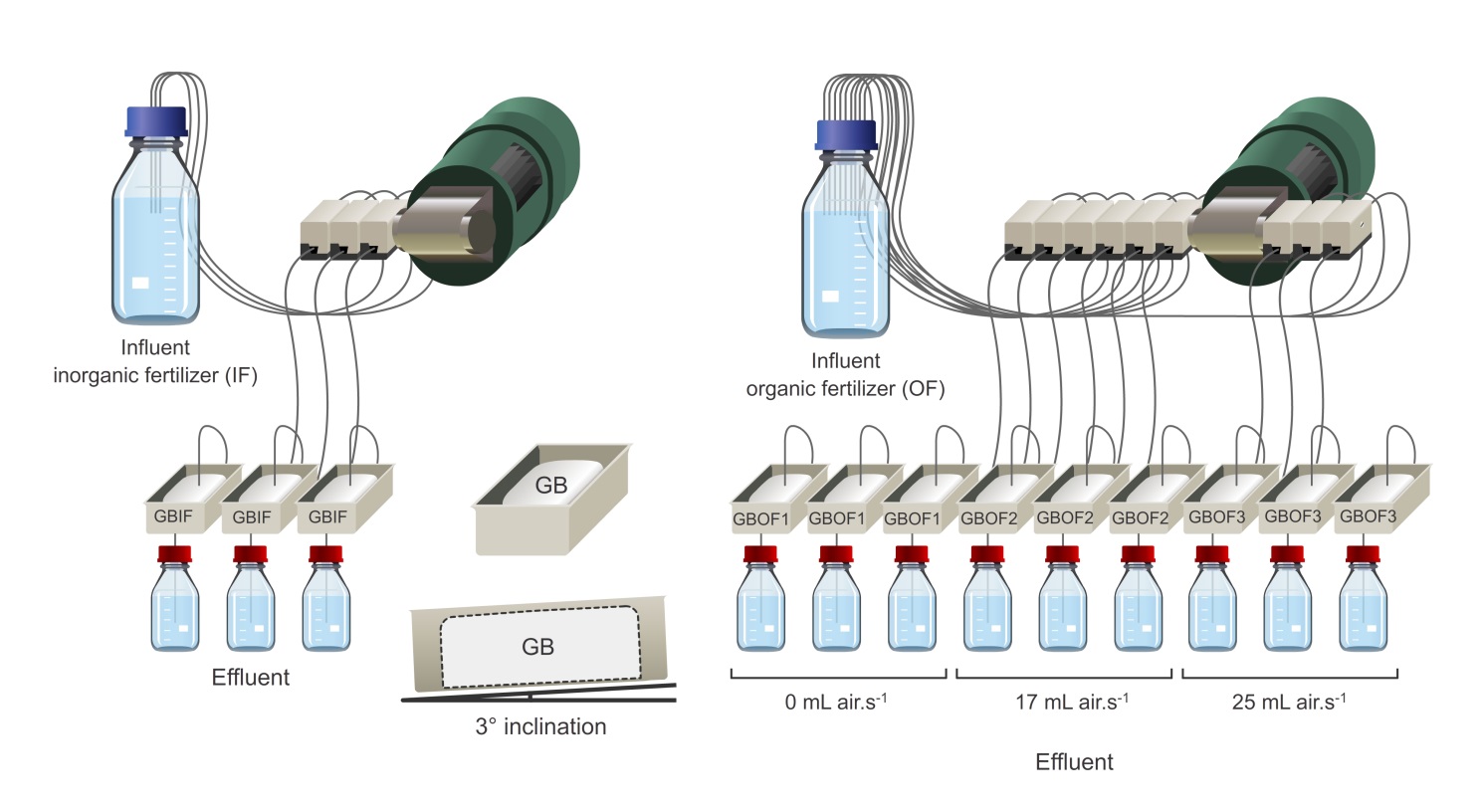


Figure 6: Experimental setup of the growing media reactor systems (GMRS). GB = growing bag with organic growing medium, IF = inorganic fertilizer and OF = organic fertilizer. Air was blown in the growing medium (respective air flow rates for GBOF1, GBOF2 and GBOF3 are indicated). The nitrogen loading rate was equal for all the systems: N_IF_ = N_OF._
